# First construction of infectious clone for newly emerging mutation porcine circovirus type 2 (PCV2) followed by comparison with PCV2a and PCV2b genotypes in biological characteristics *in vitro*

**DOI:** 10.1186/1743-422X-8-291

**Published:** 2011-06-10

**Authors:** Long J Guo, Yue H Lu, Li P Huang, Yan W Wei, Hong L Wu, Chang M Liu

**Affiliations:** 1 Division of Swine Infectious Diseases, State Key Laboratory of Veterinary Biotechnology, Harbin Veterinary Research Institute of Chinese Academy of Agricultural Sciences, No.427 Maduan Street, Nangang District, Harbin 150001, P.R. China

## Abstract

**Background:**

Porcine circovirus type 2 (PCV2), the causative agent of postweaning multisystemic wasting syndrome (PMWS), is a serious economic problem in the swine industry. Different genotypes (PCV2a, PCV2b and PCV2d) of the virus are present in the clinical cases in China, and it is necessary to elucidate the pathogenic difference among different genotypes of PCV2. In this study, four strains of different genotypes were isolated, two were ordinary strains and another two were mutation strains, which there are one and two amino acids elongation in the capsid protein (Cap) of PCV2, respectively. Representative strains of different genotypes of the virus were constructed by infectious molecular clone and biological characterization of the rescued viruses were identified *in vitro*.

**Results:**

Four PCV2 isolates (PCV2a/CL, PCV2b/YJ, PCV2b/JF and PCV2d/BDH) of different genotypes were isolated from the clinical cases of PMWS in China. Four infectious clones of PCV2 were constructed and the rescued viruses were harvested after transfection into PK15 cells. The rescued viruses were verified by nucleotide sequence analysis, morphology of the viruses and immunoperoxidase monolayer assay (IPMA). The rescued viruses propagated stably after consecutive incubation for more than ten passages, and virus propagation reached its peak 72h post infection (PI), and the virus titers were up to 10^5.7 ^TCID_50_/ml. By using neutralizing 1D2 monoclonal antibody (mAb) of PCV2, the antigen capture ELISA showed that only the PCV2a/rCL and PCV2b/rJF strains has immunoreactivity with the 1D2 mAb, however, another two rescued strains (PCV2b/rYJ and PCV2d/rBDH) do not, which indicated the antigenic difference among the rescued viruses of different genotypes. In addition, here is the first report of obtaining the newly emerging PCV2 with mutation *in vitro *by infectious molecular clone technology.

**Conclusions:**

Conclusions drawn from this study show that PCV2 has prevailing differences in genomic and ORF2 gene length and antigen in swine herds in China. Four representative clones for different genotypes were constructed and rescued, which will facilitate further studies on the pathogenic differences resulting from different subtypes of PCV2.

## Background

Porcine circovirus type 2 (PCV2) is a small non-enveloped virus with a single-stranded circular DNA genome of approximately 1.7 kb in size in the genus *Circovirus*, family *Circoviridae *[1-4]. Its genome contains at least two potentially functional open reading frames (ORFs): ORF1 (945 bp) encodes the Rep protein involved in viral replication and ORF2 (702 bp) encodes the immunogenic capsid protein [5-8]. PCV2 is generally considered to be the primary causative agent of postweaning multisystemic wasting syndrome (PMWS), which has become a serious economic problem for the swine industry worldwide. This disease is first recognized in Canada in 1997, and then subsequently identified in pigs in the USA, France, Japan, Korea and other countries [9-11]. Recently, a basic unified nomenclature for PCV2 genotypes named as PCV2a, PCV2b and PCV2c, proposed by the EU consortium on porcine circovirus diseases http://www.pcvd.net, facilitate the scientific communication and studies [12]. Genetic variation among PCV2 prevailing isolates has been reported by researchers worldwide in recent years [13-21], and we have previously demonstrated that some PCV2 with occurrence of variation or mutation (a shift of ORF2 from 702 to 705 nt for PCV2d or 708 nt for PCV2b) were prevailing in the field in China [7]. In this study, the aim was to first rescue newly emerging porcine circovirus type 2 with mutations as well as another two basic genotypes strains (PCV2a and PCV2b), with an introduction of tractable markers, respectively, by infectious molecular clone techonology. Then, biological characteristics of the four rescued PCV2 as representatives of different genotypes were identified *in vitro*, which will facilitate the further studies on the differences in pathogenicity among different genotypes of PCV2, especially the studies on the new emerging PCV2 strains with mutation.

## Materials and methods

### Cells and viruses

A porcine kidney PK-15 cell line free of PCV1 contamination that is highly permissive for PCV2 replication was maintained in a minimum essential medium (MEM) (Invitrogen, Grand Island, USA) supplemented with 5% heat-inactivated fetal bovine serum (FBS) (Invitrogen, Grand Island, USA) at 37°C under 5% CO2. PCV2 was propagated in PK15 cells as previously described [22]. Anti-PCV2-positive polyclonal swine sera and PCV2-negative swine sera were prepared by our laboratory. Strain PCV2/LG was kept in this laboratory and was selected as the control in this study.

### Virus isolation

Samples from clinical collected materials with PMWS case were determined by PCR for PCV2. To isolate the viruses, positive samples were freeze-thawed three cycles, fragmented and centrifuged. Filtered supernatants were inoculated onto PK15 cells, free of PCV1 contamination. The PK15 cells were maintained at 37°C with 5% CO2 in minimum essential medium (MEM) (Invitrogen) and 5% heat-inactivated FBS (Invitrogen). PCV2 was isolated from the culture supernatants and the isolates were then detected by PCR and an immunoperoxidase monolayer assay (IPMA) to confirm the presence of PCV2.

### Genomic amplification for the four isolated viruses by PCR

To isolate PCV2 DNA, viral isolates were freeze-thawed three times and then centrifuged. The supernatant was then used as a template for PCR amplification of the four isolated PCV2 genomes. The purified PCV2 genomic DNA was PCR amplified using the primer pairs: PCV2-F1 (920-946 nt; 5'-GTCGACGGAGGAAGGGGGCCAGTT-3') and PCV2-R1 (925-901 nt; 5'-GTCGACTGTTCTGTAGCATTCTTCCA-3'). Each primer was modified by two nucleotides to create a *Sal *I restriction enzyme site as a tractable marker (two modifications of A to G and C to G at the genomic positions 920 nt and 924 nt, respectively, underlined). PCR reactions (25 μl) contained 5 μl of KOD DNA polymerase reaction buffer (Toyobo, Osaka, Japan), 0.1 mM of each deoxynucleoside triphosphate, 0.4 μM of each primer, 1 μl (1 unit) of KOD-Plus-Ver.2 high fidelity DNA polymerase (Toyobo) and 100 ng of purified DNA. PCR was performed on a thermocycler under the following conditions: 5 min at 94°C, followed by 35 cycles of 30 s at 94°C, 30 s at 61°C and 2 min at 72°C, and a final step of 10 min at 72°C.

### Construction of recombinant plasmid including the genome of the isolates

PCR amplification of PCV2 genome was performed as previously described. Amplicon products were purified using the QIAquick PCR purification kit (Qiagen, Hilden, Germany), following the manufacturer's instructions. Prior to cloning of the purified DNA fragment, an A tail was added to the 3' terminus of the amplicon by incubation of 12.5 μl of Premix Taq Hot start version mixture (Takara, Dalian, China) with 12.5 μl of purified PCR product at 72°C for 30 min. The amplicon with an A tail was then purified using the QIAquick PCR purification kit, cloned into the pMD18 T vector system (Takara, Dalian, China) and transformed into *E. coli *TOP10 competent cells. The resulting colonies were screened according to the manufacturers' instructions. Positive colonies were detected by PCR described above, except that the first denaturation step was performed at 94°C for 10 min. Plasmid DNA was extracted using the Axygen Plasmid Miniprep Kit (Axygen, Hangzhou, China) according to the manufacturers' instructions and recombinant plasmids were identified by restriction enzyme analysis with *Sal *I. Positive plasmids from ten different colonies per strain were selected for sequencing by the commercial facility (Sangon, Shanghai, China), and both strands of the insert were sequenced at least twice, using the M13 universal primers. The sequences of the DNA fragments were then assembled using DNAMAN software (Version 5.2.2, Lynnon Biosoft, 1994).

### Constructions of infectious clone and cell transfection

To obtain an infectious DNA clone of the virus, four bacteria containing confirmed positive recombinant plasmids (pMD18-PCV2/CL, JF, YJ and BDH) were shaken for large culture after sequence verification, respectively. The recombinant plasmids were extracted using the QIAGEN Plasmid Midi Kit (Qiagen, Hilden, Germany) based on the manufacturers' instructions. The genomic DNA was separated from the plasmid DNA after digestion with *Sal *I by electrophoresis on 1% agarose gels. Viral genomes were extracted using the Axygen DNA Gel Extraction Kit (Axygen, Hangzhou, China) according to the manufacturers' instructions and self-ligation was performed with T4 DNA ligase (Takara, Dalian, China) at 16°C for 30 min. PK15 cells were seeded into six-well tissue culture plates (1 × 10^5 ^cells/well) and grown to approximately 60%-80% confluency. After one wash with OptiMEM medium (Gibco BRL, Grand Island, USA), cells were transfected with 1.2 μl of ligation DNA using Lipofectamine 2000 (Invitrogen, Carlsbad, USA) according to the manufacturers' protocol. The cells were then overlaid with complete medium after 6 hours (h) post transfection.

### Recovery of the rescued viruses and antigenic identification

Viruses with tractable genetic marker were recovered from the transfected cells for serial passage as described previously [22]. The antigenicity of rescued viruses at the fourth passage was detected 72 h by IPMA as described [23]. IPMA was used to detect antigenic activity of PCV2 positive serum to the rescued PCV2 from the fourth passage. Briefly, 96-well plates containing the rescued PCV2, PCV2/LG positive-infected and mock-infected cells as controls were fixed in 33% acetone-PBS for 20 min at room temperature and dried. PCV2 positive serum was added to rescued PCV2, PCV2/LG positive-infected and mock-infected cells, respectively, and then incubated at 37°C for 1h. After the unbound antibodies were washed three times with PBS, a 1:3,000 dilution of HRP- conjugated Protein A (Invitrogen, Carlsbad, USA) was added and the samples were incubated for 1h at 37°C. After washing, color development was carried out with 3-amino-9-ethylcarbazole and hydrogen peroxide in 0.05M acetate buffer (pH5.0) for 30 min at 37°C. The reaction was terminated by removal of the substrate. The plates were examined under an inverted light microscope. The IPMA was performed in triplicate.

### Genomic DNA cloning and sequencing of rescued viruses

The four full-length PCV2 PCR products amplified from the DNA extracted from the rescued cells were inserted into the pMD18-T vector as the method described above. Positive plasmids from ten different colonies per strain were selected for sequencing by the commercial facility (Sangon, Shanghai, China), and both strands of the insert were sequenced at least twice, using the M13 universal primers as mentioned above. The sequences of the DNA fragments were then assembled using DNAMAN software (Version 5.2.2).

### Differentiation of rescued viruses with tractable marker from wild-type

A pair of primers was designed to amplify the fragment of both sides of *Sal *I sites. The forward primer PCV2-F2 (56-80 nt): 5'-CAGCAAGAAGAATGGAAGAAGCGGA-3' and the reverse primer PCV2-R2 (1112-1088 nt): 5'-CCAGGACTACAATATCCGTGTAACT-3' were used for PCR amplification, which generated a fragment of 1057 bp using either the tractable marker viruses or wild-type viruses DNA as template. PCR products from the rescued viruses with tractable marker were digested with *Sal *I to generate two fragments of 865 and 192 bp. PCR products from the wild-type viruses could not be digested with *Sal *I, thus remaining as 1057 bp fragments.

### Morphological observations of the rescued viruses

The virus cultures were mixed with a 1:100 dilution of PCV2-specific antibodies for incubation overnight at 4°C, and then centrifuged at 14,000 ×g for 30 min. The immune complexes containing viral particles were harvested and morphological characteristics of the virus were observed using electron microscopy.

### PCR-RFLP analysis of rescued virus

PCR was performed as described above and PCR products were digested with 1 U of the restriction enzymes *Fba *I and *Acc *I (Takara, Dalian, China), in a volume of 30 μl for 4 h at 37°C. The digestion products were then separated by electrophoresis on 1% agarose gel.

### Viral titration for rescued viruses for consecutive passages

The viral cultures of different passages were serially diluted 10-fold in MEM medium supplemented with 2% FBS and antibiotics. Each rescued virus dilution was inoculated into four separate wells containing 100 μl PK15 cells in suspension. Virus titers in cell cultures for each passage were determined by a microtitration infectivity assay and recorded as TCID_50 _/ml by using the Reed-Muench method. Briefly, cells were prepared in 96-well plates and inoculated with virus suspensions (100 μl/well), which were prepared by serial 10-fold dilution. After absorption for 1 h at 37°C, the liquids in the wells were removed, and MEM with 2% FBS was added to the wells. Plates were incubated for an additional 72 h at 37°C. After the media were removed, the cells were fixed with a 33% acetone-PBS solution for 20 min at room temperature and air-dried. Virus titers were determined by the presence of a visible positive cell by the IPMA method using PCV2-specific antibodies as previously described [23].

### Kinetics of the rescued viruses

The rescued viruses from the 10^th ^passage were inoculated into the six-well tissue culture plates with PK15 cells suspension and a mock-infected cell well was used as a control. After inoculation at 0 h, 12 h, 24 h, 48 h, 60 h, 72 h, 84 h and 96 h, the cells for each rescued PCV2 were fixed with a 33% acetone-PBS solution for 20 min at room temperature and air-dried, respectively. Then, the characteristics of multiplication for the rescued viruses were evaluated as the IPMA method previously described [23].

### Antigenic analysis of the four rescued viruses by capture ELISA using mAb 1D2

MAb 1D2 against PCV2 Cap protein has been prepared as previously described [23]. The mAb 1D2 is against a conformational epitope and demonstrates neutralization activity to PCV2 by a neutralization test (data not shown). For the antigen capture ELISA, 96-well microtiter plates were coated with swine anti-PCV2 positive sera (1:100 dilution) in 0.1 M carbonate buffer (pH 9.6) at 4°C overnight, and were then blocked with 5% skimmed milk for 3 h. After blocking, the plates were washed three times with PBST (PBS with 0.1% Tween 20). In the binding assay, plates were incubated with the culture supernatant of the four rescued viruses (10^4.5^TCID_50_/ml), respectively, at 37°C for 1 h, followed by three washes with PBST. Bound rescued viruses were detected with horseradish peroxidase (HRP)-coupled 1D2 mAb against PCV2 Cap. Then, 100 μl of 2,2'-azino-bis(3-ethylbenzthiazoline-6-sulphonic acid (ABTS) was added and after 30 min the reaction was stopped with 2 M H_2_SO_4 _and the absorbance at 405 nm was measured using an ELISA plate reader (Bio-Rad, Hercules, CA, USA).

## Results

### Characteristic of the cell isolates from different genotypes

**Figure 1:**
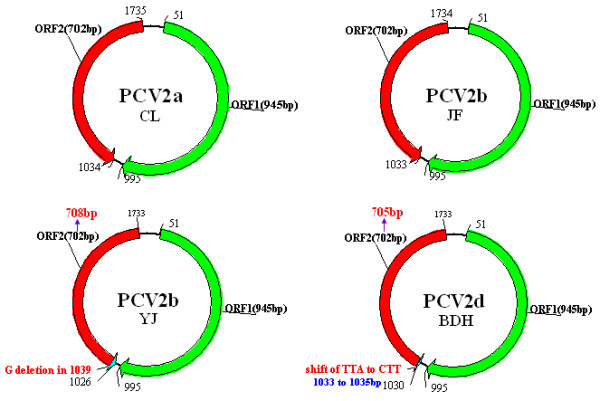
**The schematic diagram of PCV2 strains of different genotypes in detail**. Analysis of the ORF2 gene in PCV2b/YJ indicated a one-base deletion at position 1039 in the genome, resulting in an ORF2 gene of 708 nt. For PCV2d/BDH strain, a shift from TTA to CTT in the genomic sequence resulted in the stop codon mutation (from UAA to AAG) in the ORF2 (at the anti-sense chain of the genomic sequence of PCV2), being an ORF2 gene of 705 nt in another stop codon.

Four representative strains were isolated from clinical collected materials with multisystemic lesions and clinical signs of PMWS following the verification of nucleic acid sequencing, an antigen-based IPMA as well as immune electron (data not shown). The genotypes of the genomic sequences of the four isolated strains were designated according to the method of Grau et al. [12]. PCV2/CL strain belonged to the PCV2a genotype; PCV2/YJ, PCV2/JF strains belonged to the PCV2b genotype and PCV2/BDH strain belonged to another new genotype named as PCV2d [7]. The four representative strains were named as PCV2a/CL, PCV2b/JF, PCV2b/YJ and PCV2d/BDH according to the genotype nomenclature. The four sequences were submitted to the GenBank database and assigned accession numbers HM038034, HM038022, HM038032 and HM038017, respectively. The schematic diagram for the four representative strains of PCV2 different genotypes is illustrated in Figure 1.

### Obtaining of PCV2 infectious clone

The infectious clone was obtained through the self ligation of the genomic DNA followed digestion of recombinant plasmid by *Sal *I, gel extraction and ligation at the created *Sal *I site by T4 DNA ligase. PK15 Cells were transfected with 0.8 μg of self ligation DNA using Lipofectamine 2000 (Invitrogen) according to the manufacturer's protocol.

### Identification of the rescued viruses

**Figure 2:**
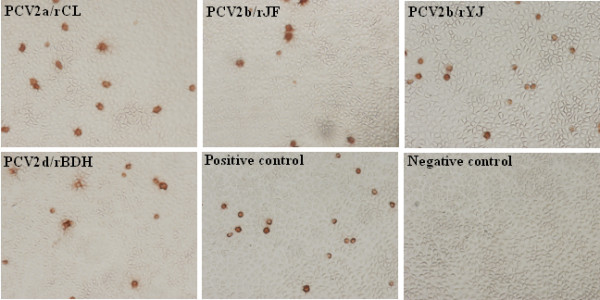
**Identification of four cloned strains of different PCV2 genotypes (PCV2a/rCL, PCV2b/rJF, PCV2b/rYJ and PCV2d/rBDH) including two new emerging mutants by IPMA**. Each of the four rescued strains reacted with swine anti-PCV2 positive sera. PCV2/LG strain was included as a positive control and PK15 cells were used as negative control.

PK15 cells were transfected with the infectious clone of the virus and four viruses named PCV2a/rCL, PCV2b/rJF, PCV2b/rYJ and PCV2d/rBDH as representatives of different genotypes were successfully rescued by infectious clones after transfections following the identification of sequencing, serological detection (Figure 2). Based on the sequencing, the cloned viruses were completely the same as the parental strains in nucleic acid sequence. Viral antigens were detected by IPMA using PCV2-specific serum. As shown in Figure 2, the virus-infected cells stained brownish-red, while control cells transfected in parallel as the mock control showed no staining. The results revealed the presence of recovered PCV2 as determined by serology.

### Sequencing of the rescued viruses

**Figure 3:**
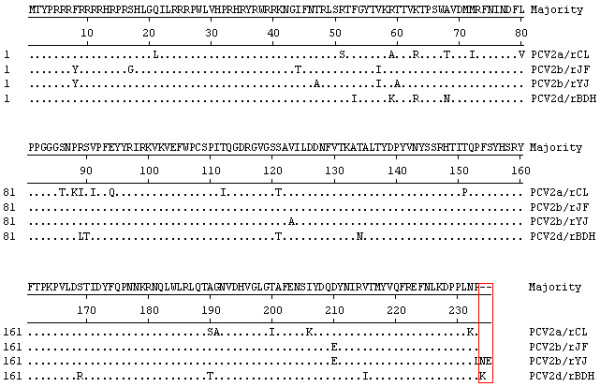
**Sequence alignment of the deduced amino acid (aa) sequences of the capsid proteins (encoded by ORF2) of the four cloned strains of PCV2**. Mutations at the C terminus of the capsid protein in the four rescued viruses are shown, resulting in elongation of the ORF2 by one lysine residue (for PCV2d/rBDH) or asparagine and glutamic acid residue (for PCV2b/rYJ), compared with the wild-type sequence (red box).

**Figure 4:**
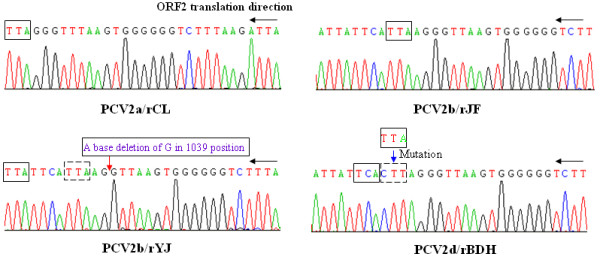
**Analysis of the precise site changes contributed to mutations of Cap protein in the corresponding position in the genome level**. Based on sequencing results of the four rescued PCV2, PCV2b/rYJ showed, and PCV2d/rBDH showed an elongation of one amino acid (Lys) compared with the PCV2a/rCL isolations. Analysis of the genomic sequencing in the four rescued strains indicated that a one-base deletion at position 1039 in the genome of strain PCV2b/rYJ (refer to the red arrow) resulted in an elongation of two amino acids (Asn and Glu) in ORF-encoded Cap protein and a codon mutation from TTA to CTT (marked in black dash box) in the genomic level of PCV2d/rBDH led to the stop codon mutation from UAA to AAG, being in an amino acid elongation by the next stop codon UGA (reverse complement of TCA, marked in black box), compared with that of the PCV2a/rCL. Black arrow and black box represent ORF2 translation and ORF2 stop codons of the four rescued viruses, respectively.

The four full-length PCV2 PCR products amplified from the DNA extracted from the rescued cells were inserted into the pMD18-T vector as the method described above. Two bands of the expected sizes were obtained for each strain, indicating that the genomes of the four rescued PCV2 strains had been successfully cloned into the vectors. The four sequences of the cloned viruses were exactly the same as the parental viruses, respectively. Based on the analysis of the four ORF2-encoded Cap protein sequences, important mutations located in the C terminus of the Cap protein are indicated by the red circle in Figure 3. PCV2b/rYJ showed an elongation of two amino acids (Asn and Glu), and PCV2d/rBDH showed an elongation of one amino acid (Lys), compared with the PCV2a/rCL isolations. Analysis of the ORF2 gene in the four rescued strains indicated a one-base deletion at position 1039 in the genome of strain PCV2b/rYJ (Figure 4), resulting in an ORF2 gene of 708 nt. While for PCV2d/rBDH, a stop codon mutation in the ORF2 resulted in an ORF2 gene of 705 nt (Figure 4). ORF2 gene was elongated by one or two codons due to the deletion and mutation mentioned above. The basic characteristics for the rescued viruses are referred to in Table 1.

**Table 1:** Characteristic of rescued viruses including two mutation strains from different genotypes

Items	Name of rescued viruses			
	
	PCV2a/rCL	PCV2b/rJF	PCV2b/rYJ	PCV2d/rBDH
GenBank No	HM038033	HM038022	HM038032	HM038017
Genotype	PCV2a	PCV2b	PCV2b	PCV2d
Year of isolation	2008	2008	2008	2008
Genome length (nt)	1768	1767	1766	1767
ORF2 length (nt)	702	702	708	705
ORF1 length (nt)	945	945	945	945
Number of amino acid*	0	0	2 (N and E)	1 (K)

Note: * represent the elongation number of amino acid encoded by ORF2 compared with classical PCV2 strains with 233 aa in ORF2-encoded Cap protein.

### Differentiation of rescued viruses with tractable marker from wild-type parental viruses

**Figure 5:**
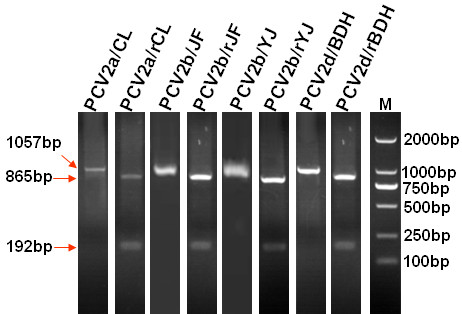
**Differentiation of the rescued viruses with tractable marker from their parental wild-type viruses**. *Sal *I digestion of PCR products of rescued viruses with tractable marker generated two fragments of 865 and 192 bp; *Sal *I digestion of PCR products of parental wild-type viruses generated only one fragment of 1057 bp. The names of the rescued viruses and parental wild-type viruses are indicated at the corresponding lane above the figure.

The rescued viruses with tractable marker and their parental wild-type viruses were differentiated by *Sal *I digestion of PCR products from amplification of differentiation primers. The size of the PCR products amplified using primers PCV2-F56 and PCV2-R1112 were 1057 bp, with no *Sal *I site in the parental wild-type viruses. The genomes of the rescued viruses with tractable marker contained the *Sal *I site, and could therefore be digested into two fragments of 865 and 192 bp (Figure 5). Therefore, the rescued viruses with tractable marker could be differentiated from the parental wild-type viruses by the method mentioned above.

### Morphological observations by electron microscopy

**Figure 6:**
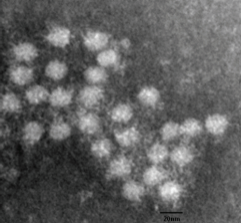
**Immune electron micrograph of negatively stained rescued PCV2 particles (bar = 20nm)**. The diameter of particles was about 17 nm corresponding to the size reported formerly.

The individual cloned virus particle had an approximate diameter of 17 nm, as determined by electron microscope examination (Figure 6).

### Characterization of the rescued viruses by PCR-RFLP

**Figure 7:**
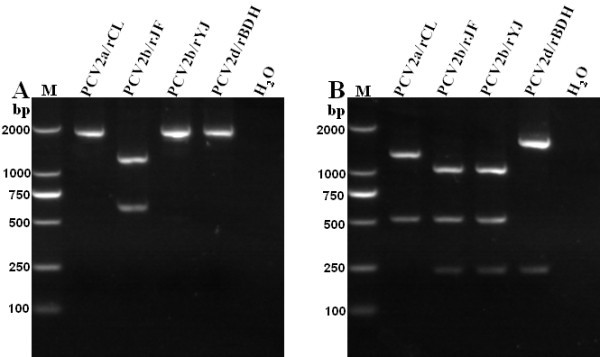
**PCR-RFLP profiles of the amplified rescued PCV2 genomic DNA after digestion with restriction enzyme of *Fba *I (A) and *Acc *I (B)**. Rescued names are indicated above the corresponding lanes. Panel A shows the *Fba *I digest of the PCR amplification fragments and restriction fragments are evident at 1165 and 602 bp for PCV2b/rJF, the DNA of other rescued strains (PCV2a/rCL, PCV2b/rYJ and PCV2d/rBDH) remains undigested. Panel B shows the *Acc *I digest of the PCR amplification fragments and restriction fragments are evident at 1257 and 511 bp for PCV2a/rCL; 1532 and 235 bp for PCV2d/rBDH; and 1021 511 and 235 bp for PCV2b/rJF and PCV2b/rYJ. The four rescued strains of different genotypes as well as mutation rescued viruses can be differentiated by the method via combination use of *Fba *I and *Acc *I.

Genomic PCR amplification products from the four isolated PCV2 rescued strains were characterized by digestion with restriction enzymes *Fba *I and *Acc *I, and analysis of the resulting electrophoresis patterns. The four cloned viruses were differentiated from each other by digestion with the restriction enzyme *Fba *I and *Acc *I (Figure 7). The results indicated that differences in nucleic acid level existed among the four rescued viruses of different genotypes and an effective and simple method could be established to differentiate the four rescued PCV2 of different genotypes via PCR-RFLP using two restriction enzymes.

### Viral titration for serial passages

**Figure 8:**
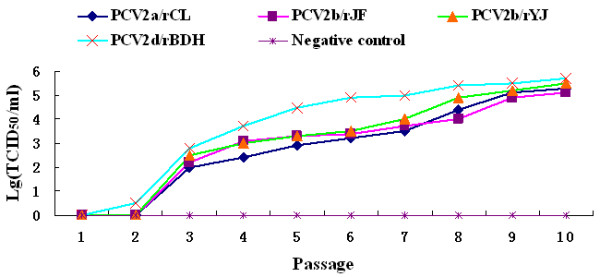
**Growth curves of the cloned virus strains after a series of passages**. The virus titers increased with the number of passages. The virus titers strikingly increased since the second passage and reached up to 10^5.7 ^TCID_50_/ml at the 10^th^ passage.

The rescued viruses were continuously cultivated in PK15 cells for 10 passages. The virus titer increased with the number of passages. The virus titers strikingly increased during the second passage and reached up to 10^5.7 ^TCID_50_/ml at the 10^th ^passage (Figure 8). The *Sal *I site as the genetic marker in the genome of the rescued viruses was stable after 10-serial passages *in vitro*.

### Kinetics of the rescued viruses

**Figure 9:**
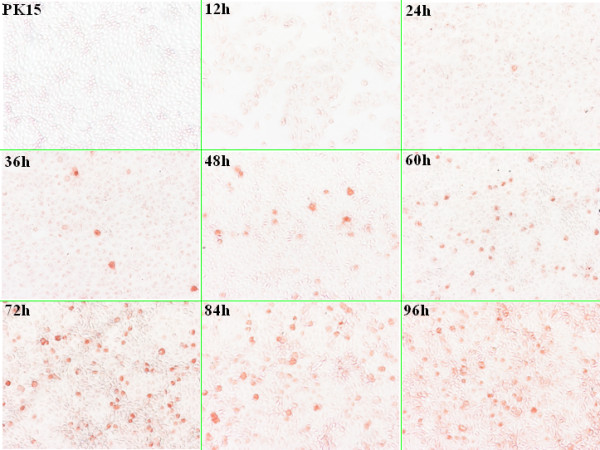
**Cell culture kinetics of the rescued viruses in different time by IPMA**. Positive-infected cells first occurred at 24 h PI. followed by peak time of positive-infected cells at 72 h PI, and then plasmatorrhexis was present because of the large multiplication of viruses, which led to disseminated distribution of the positive-infected cells.

Based on the studies of the Kinetics on cell culture of the rescued viruses, the result indicated that positive-infected cells first occurred at 24 h post inoculation followed by a peak time of positive-infected cells at 72 h post inoculation. 72 h later, plasmatorrhexis was present because of the large propagation of viruses, which led to disseminated distribution of the positive-infected cells (Figure 9).

### Differences in antigen among the four rescued viruses

**Figure 10:**
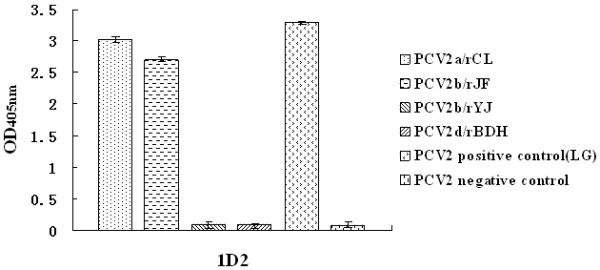
**Reactivity of the four rescued viruses with mAb 1D2**. PCV2a/rCL and PCV2b/rJF showed strong reactivity with mAb 1D2 and the remaining rescued viruses (PCV2b/rYJ and PCV2d/rBDH) are not recognized by mAb 1D2, which indicates that the ORF2 mutation of the rescued viruses results in antigenic changes in the PCV2 Cap protein.

Antigen capture ELISA using mAb 1D2 showed that only PCV2a/rCL and PCV2b/rJF rescued virus had strong reactivity with this mAb, while the other two rescued viruses (PCV2b/rYJ and PCV2d/rBDH) did not. The result indicated that mutation of the ORF2 gene resulted in antigenic changes within the PCV2 Cap protein for another three cloned viruses (Figure 10).

## Discussion

PCV2 has been recognized as the primary causative agent of PMWS, an economically harmful wasting disease of young pigs [24]. Disease caused by PCV2 infection has increased in severity in China in recent years, resulting in increased morbidity and mortality in swine and significant economic losses in the swine industry [7]. More and more studies on the genetic variation about PCV2 have been increasingly reported at home and abroad in recent years and some new emerging PCV2 mutants with one or two amino acids elongation in ORF2-encoded capsid protein compared with that of the ordinary PCV2 were already present in clinical swine farms suffering from PCV2 infection [18]. In this study, four rescued PCV2 strains were constructed as representatives prevailing in China corresponding to the different genotypes. In addition, this is the first report of obtaining the newly emerging PCV2 mutation strains (PCV2b/rYJ and PCV2d/rBDH) with a *Sal *I tractable differentiation marker from wild type parental PCV2 via infectious molecular clone worldwide, although infectious clone only for common infectious PCV2 has been already rescued by Fenaux et al. in another different way [25]. The stability of the rescued mutation PCV2 was verified by successful 10-serial passages in PK15 cells followed by IPMA and DNA sequencing conformation after a series of passage (data not shown). It could be speculated that the mutants could be the predominant genotypes that are able to evade the immune defense of the host, which could cause much more severe diseases to the swine production industry. Therefore, on the one hand this study was helpful to compare the pathogenicity of different genotypes as well as the mutation PCV2, and on the another hand it helped facilitate further studies on the mechanism of pathogenicity for mutation PCV2.

Pure different genotypes PCV2 together with mutants were obtained by infectious clones, which avoid the interference of other viruses as contaminant during animal experiments. Based on the animal experiments with pure PCV2 particles, more scientific and reliable results on the pathogenic differences among the different genotypes as well as the pathogenic changes resulting from PCV2 mutations could be illustrated. First constructions of the PCV2 mutation strains will facilitate the further investigations on pathogenecity caused by variations as well as early prevention and control ahead of prevailing of newly emerging PCV2 with mutation.

## Conclusion

Taken together, four rescued PCV2 of different genotypes including two newly emerging PCV2 mutation strains were obtained in the study. Two newly emerging PCV2 with mutation were PCV2b/rYJ(ORF2 = 708 bp)and a novel genotype PCV2d/rBDH(ORF2 = 705 bp), respectively. Here is the first report of obtaining newly emerging mutation PCV2 with an introduction of *Sal *I as a tractable marker and confirmation of genetically stability in serial passages *in vitro*. In addition, mutation of the ORF2 gene resulted in antigenic changes within the PCV2 Cap protein by using the mAb 1D2 in capture ELISA assay. The results will facilitate further studies on differences in pathogenicity among different genotypes PCV2 as well as newly emerging mutation PCV2 prevailing clinically in China.

## Competing interests

The authors declare that they have no competing interests.

## Authors' contributions

LJG organized the whole process, took part in all the experiments and wrote the manuscript. CML designed the whole project. YHL participated in the clinical materials collection; YWW made great contribution to the virus isolation; LPH carried out the cell culture and preparation of mAb. HLW participated in detection of capture ELISA method. All authors read and approved the final manuscript.
